# The Elastic Ligature

**Published:** 1874-01-01

**Authors:** 


### THE ELASTIC LIGATURE.

*From the British Medical Journal, Nov. 29th, 1873.*

ON the 21st instant, Sir Henry Thompson demonstrated, for the first time in England, a surgical procedure which has been practiced for some time past by Professor Dittel, of Vienna. It consists in substituting an innocent-looking elastic thread for the formidable array of knives, tourniquets, artery-forceps, and other paraphernalia with which the surgeon ordinarily approaches the patient. Before proceeding to perform the operation, Sir Henry related the curious accident by which Professor Dittel was first led to appreciate the extraordinary results which may be produced by the slight, yet continuous, pressure of a simple elastic thread. He was called to see a girl about eleven years of age, who was suffering from acute and severe, but somewhat anomalous brain-symptoms. The case was altogether obscure; the girl seemed in other respects healthy, but could give no account of herself—she was, in fact, at the point of death—nor could any satisfactory history be obtained from her friends. The attack soon proved fatal, and Professor Dittel made a necropsy. It was then found that the India-rubber band of the hair-net which she was wearing had ulcerated through the whole thickness of the calvarium, and had set up meningitis. On further inquiry, it was ascertained that the girl, having been constantly scolded by her stepmother on account of the untidy state of her hair, had, about three weeks before her illness, purchased an ordinary hair-net, and the elastic thread of this net, tied around the head, and worn day and night, had, in less than a month, cut through skin and bone and penetrated to the brain; and this apparently without causing any pain to the patient.

Professor Dittel at once proceeded to reduce to practice the idea suggested to him by this unfortunate accident. He first applied it to a case of nævus of the scalp in a child; then, finding that the plan quite answered his expectations, he applied it to the removal of the testicle, penis, etc., and finally to the amputation of limbs. He has now performed, by means of the elastic ligature, a large number of operations of all kinds, including five amputations of limbs. It is not understood, however, that he proposes to apply his method to the performance of the larger amputations; these were done rather with the view of testing the capabilities of the process. The time required for the completion of an operation varies according to the amount and density of the tissues which have to be divided, *e. g.*, for the separation of the mamma from eight to twelve days.

The chief advantage which Dr. Dittel asserts this plan to possess is, that patients so operated on are less liable to pyæmia than those treated in the ordinary way. He bases this assertion on the experience of the numerous cases referred to above. Remembering also what a morbid dread of the knife many nervous patients have, the depressing mental effects of an operation may often be greatly diminished. Lastly, the operation itself is absolutely bloodless.

Among the operations for which it is admirably adapted may be especially mentioned *fistula in ano*, which Dr. Dittel now invariably treats in this way. One end of the India-rubber thread is passed in the eye of a probe up the sinus into the bowel, then caught, brought out at the anus, and tied; it cuts out in a few days.

The patient on whom Sir Henry Thompson operated was a stout, middle-aged woman, who was suffering from an ulcerating fibro-cystic tumor (cystic sarcoma) of the right breast. She had had a lump in the breast for twenty years, but it caused her little inconvenience till two years ago, when it began to enlarge rapidly, and finally the skin over it gave way. At the time of the operation the tumor was of the size of a large orange, and somewhat pendulous, the breast itself being wasted; it was crowned by a large, sloughy, fungating ulcer. The ligature used was tublar, about one-twelfth of an inch in diameter, the calibre of the tube being about one-third of this. A large nævus needle was threaded with this and with a piece of twine (the use of which was explained afterwards) and passed under the base of the tumor; the elastic was then cut, the needle withdrawn, and the halves of the pedicle tied separately.

Sir Henry Thompson remarked, after the operation, that, although this was a very suitable case for the method adopted, it was not a severe test. The only accident that could happen was the snapping of the elastic when stretched; in that case, another length was tied to the twine, which had been passed under the tumor with the elastic and drawn by it along the track of the needle; otherwise, the twine was removed as soon as the ligatures had been properly tied. The best way to avoid the occurrence of this accident was always to use freshly-prepared elastic; if kept only a month, it was very liable to become brittle.

The skin over the tumor should be tightened just before tying, so that as little as possible might be included. Sir Henry added that, as that was the first time he had operated by that method, he had been anxious to comform in all respects to the practice of Dr. Dittel; but he thought that, another time, he should be disposed to make a superficial incision through the skin along the course of the ligature, so that it would be in a groove, and would not be liable to slip. He thought, also, that this would obviate the pain which Dr. Dittel said that patients sometimes experienced during the first two or three hours after the operation; in most cases, however, the pain was slight. This patient complained of pain, apparently not very severe, for about twenty minutes after she recovered from the chloroform. Dr. Dittel's paper in the *Allegemeine Wiener Medizinische Zeitung*, 1873, has furnished very full details respecting this mode of treatment.

